# Determination of activities of ^210^Pb in Slovak tobacco and cigarettes: a study on radiological risks

**DOI:** 10.1007/s00411-024-01098-9

**Published:** 2024-12-12

**Authors:** Silvia Dulanská, Veronika Demovics Silliková, Zuzana Goneková, Michaela Ticháková, Klára Gebeová, Michal Trnka, Daniel Kosnáč, Ján Pánik

**Affiliations:** 1 Slovak Medical University, Limbová 12, 833 03 Bratislava, Slovakia; 2 Institute of Inorganic Chemistry, Slovak Academy of Sciences, Dúbravská cesta 9, 845 38 Bratislava, Slovakia; 3 Institute of Laboratory Research on Geomaterials, Comenius University, Mlynská dolina, Ilkovičova 6, 842 15 Bratislava 4, Slovakia; 4 Faculty of Medicine, Institute of Medical Physics and Biophysics, Comenius University Bratislava, Sasinkova 2, 813 72 Bratislava, Slovakia

**Keywords:** ^210^Pb, Tobacco, Extraction chromatography, Radioactivity, Annual effective doses

## Abstract

This study investigates the ^210^Pb activity concentrations in tobacco and cigarettes available in Slovakia, utilizing two specific extraction methods including the Sr Resin sorbent used in extraction chromatography, and the AnaLig Sr01 sorbent, which operates based on molecular recognition principles. The findings revealed significant variations in ^210^Pb activity concentrations, with concentrations ranging from 13.3 to 33.8 mBq/g in tobacco, and from 16.8 to 28.5 mBq/g in cigarettes. The average ^210^Pb activity per cigarette was 14.4 mBq ± 1.7 mBq. Annual effective doses for smokers were calculated, with values for tobacco ranging from 27.9 to 126.7 µSv and for cigarettes from 25.5 to 115.7 µSv. The study highlights the importance of comparing these two methods to ensure an accurate assessment of ^210^Pb exposure and evaluation of radiological risks associated with smoking in Slovakia.

**Supplementary Information:**

The online version contains supplementary material available at 10.1007/s00411-024-01098-9.

## Introduction

The long-term trend of decline in smoking in Slovakia reversed during the COVID-19 pandemic, with an increase of 21.2% compared to the pre-pandemic period. In 2022, Slovakia saw the sale of 7.4 billion cigarettes and tobacco products, indicating a 1.3% increase compared to 2021 and signaling a return to a growth trend (Nemocnica Zvolen 2023). According to the World Health Organization (WHO), tobacco use significantly impacts public health in Slovakia, contributing to thousands of smoking-related deaths annually (World Health Organization 2021).

Globally, this corresponds to approximately four million casualties, with health experts projecting a potential increase to ten million by 2030. Smoking induces chronic inflammation of the bronchi and contributes to the development of chronic obstructive pulmonary disease in up to 80% of smokers. It is also a significant factor in the onset of heart and vascular diseases, heart attacks, ischemic heart diseases, strokes, and lower limb ischemia, and plays a role in the development of peptic ulcer diseases. Additionally, smoking is associated with an increased risk of cancer affecting areas such as the mouth, larynx, esophagus, stomach, and other neoplastic diseases (Public Health Office of the Slovak Republic 2023).

Tobacco contains trace amounts of radioactive isotopes from the uranium and thorium series (^210^Pb, ^210^Po, and ^226^Ra), which are recognized as radioactive carcinogens. The radionuclide ^210^Pb is particularly important to study, because it serves as the parent of ^210^Po.^210^Po is known for its extreme toxicity and is considered one of the most dangerous alpha emitters, with high radiotoxicity that can be lethal at masses as low as one microgram (BMJ 2007).

The presence of ^210^Po in the lungs can result from two primary sources: it can be formed through the decay of ^210^Pb that has accumulated in lung tissue, or it can be directly absorbed from inhaled cigarette smoke. The long half-life of ^210^Pb (approximately 22.2 years) allows for sustained emission of alpha radiation, potentially leading to lung cancer, particularly in long-term or former smokers (Marshall 2024). Smoking and the use of tobacco products increase the intake of and radiation dose due to naturally occurring radionuclides, making them significant contributors to lung cancer. According to research published in Nicotine & Tobacco Research, long-term exposure to ^210^Po from smoking is estimated to cause 120–138 lung cancer deaths annually per 1000 regular smokers (Karagueuzian et al. 2012).

Many researchers have explored the presence of such radionuclides in tobacco. Among the 250 known harmful chemicals in tobacco smoke at least 69 can cause cancer. These carcinogenic chemicals include polonium and lead (NCI 2024; CDS 2024).

Lead is recognized as an exceptionally harmful metal with documented detrimental effects on various physiological systems, including the liver, kidneys, nervous system, bone density, white and red blood cells, fetal growth and development, diabetes mellitus and cardiovascular health (Harrison and Alpha Laxen 1984; Ratcliffe 1981). Inhalation is the dominant pathway for lead exposure among workers in industries that produce, refine, use or dispose lead and lead compounds. Workers can incorporate as much as 400 µg of lead during an 8-h shift, in addition to the 20–30 µg/day incorporated from food, water, and ambient air. Significant intake may also occur from ingestion of large amounts of inhaled particulate material. Additionally, lead is present in tobacco, indicating the potential for exposure to this heavy metal through tobacco consumption. The mean lead content in filter-tipped cigarettes produced between 1960 and 1980 was 2.4 µg/g. Approximately 5% of this lead may be inhaled; the remainder occurs in the ash and side-stream smoke (United Nations Environment Programme 1995; Mussalo-Rauhama et al. 1986). The WHO suggests that 2–6% of the lead content in cigarettes is inhaled by the smokers (Hossain et al. 2018; WHO 1989). Individuals who smoke 20 cigarettes daily are estimated to inhale 1–5 µg of Pb. In 1989, the WHO reported that around 2–6% of the concentration of lead accumulates in the lungs of smokers through cigarette use (WHO 1989).

A transfer of 46–60% of lead from tobacco to cigarette smoke was reported leading to a significant accumulation of lead in the lung tissues of smokers compared to non-smokers (Pinto et al. 2017). In a recent study, the mean amount of lead in the breath stream of smokers ranged from 1.55 to 3.51 μg, with an average value of 2.41 μg (Janaydeh et al. 2019). It has been known since 1964 that cigarette smokers face significantly higher radiation doses from ^210^Po and its parent ^21^⁰Pb (Persson and Holm 2011). Consequently, heavy smokers may receive radiation exposure with estimates ranging from 100 to 160 µSv/y (Skwarzec et al. 2001) (Skwarzec et al. 2001). The radioisotope ^210^Pb is the parent radioisotope of ^210^Po. The presence of ^210^Po in the lungs may result from the presence and subsequent decay of ^210^Pb (physical half-life: 22 years) ^210^Po, in turn, is an alpha emitter (physical half-life: 138 days). Being continuously produced due to the decay of ^210^Pb, ^210^Po is responsible for the sustained emission of alpha radiation persisting for many years even after smoking cessation. This may provide one of the potential explanations for lung cancer development, particularly among former smokers (Zagà et al. 2011). Pharmacological treatments that enhance bronchial mucociliary clearance might indeed lower the levels of ^210^Pb and ^210^Po in the lungs, thereby significantly reducing the risk of lung cancer in current smokers and especially in former smokers (Zagà et al. 2011).

When the time interval between the harvesting of tobacco leaves and cigarette production exceeds two years, ^210^Po and ^210^Pb in the cigarette tobacco reach a state of radioactive equilibrium with their activities being practically equal. In such instances, the ^210^Po/^210^Pb activity ratio in the examined samples is close to 1 (Savidou et al. 2006). However, in cases of shorter tobacco storage, their activities are not generally equal. In these situations, the ^210^Po/^210^Pb activity ratio depends on their ratio at the time of harvest and the duration of storage. A common commercial cigarette contains approximately 0.6–1 g of tobacco on average (Savidou et al. 2006).

In the present study, the primary objectives were twofold: first, to compare two methods of extraction of ^210^Pb (using the molecular recognition sorbent AnaLig Sr01 and the extraction chromatography sorbent Sr Resin); and second, to estimate the annual effective doses affecting smokers in Slovakia.

## Materials and methods

### Reagents and materials

The methods used in the present study for analyzing ^210^Pb in tobacco and cigarettes involved the use of a molecular recognition technology resin, AnaLig Sr01 (100–150 μm, IBC Technology, USA), and the Sr resin (50–100 μm, TRISKEM International, France). A ^210^Pb standard (1010 Bq/g with a relative uncertainty of 1.1%) was provided by the Czech Metrology Institute (Prague, Czechia) with the reference date for all radioisotopes set to September 18, 2015.

The equipment used included chromatographic columns for Sr resin sorbent (diameter: 0.5 cm; length: 15.5 cm) and chromatographic columns for AnaLig Sr01 sorbent (diameter: 1.5 cm; length: 15.5 cm). A liquid scintillation cocktail Ultima GOLD AB (PerkinElmer), a liquid scintillation spectrometer TRI CARB 3100TR (PerkinElmer), as well as an atomic absorption spectrometer AAS 1100 and an atomic absorption spectrometer AAnalyst 400 (PerkinElmer), were used.

### Calibration of the TriCarb 3100 TR liquid scintillation detector

**Fig. 1:**
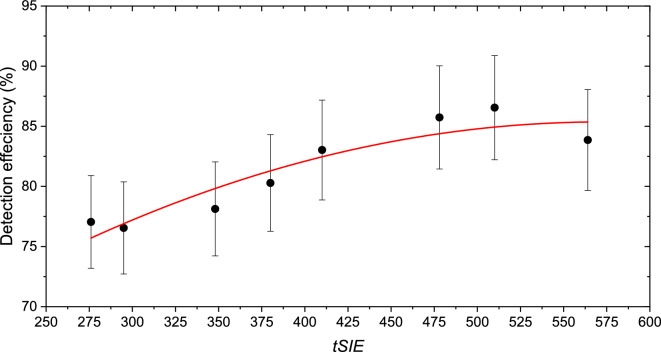
Dependence of the ^210^Pb detection efficiency with 5% of uncertainty on the transformed spectral index of the external standard (tSIE) using the liquid scintillation spectrometer TRI CARB 3100TR

The instrument used for the ^210^Pb measurements was calibrated using a certified ^210^Pb solution (in equilibrium with ^210^Bi) provided by the Czech Metrology Institute. The quench curve of the instrument was established by adding varying amounts of tetrachloromethane (ranging from 3 to 500 mg) to each sample. The quenching parameter, transformed spectral index of external standard (*tSIE*), was determined using an external ^133^Ba source with *tSIE* values ranging from 276 to 564 (Fig. 1). A mixture of 10 mL of aqueous solution and 10 mL of scintillation cocktail (Ultima GOLD AB) was added to the solutions. A measurement window in the range of 50–700 keV was selected based on spectrum evaluation for the measurement of ^210^Pb in equilibrium with ^210^Bi.

The measurement of ^210^Pb using a liquid scintillation detector, via the ingrowth of ^210^Bi, could be accurately completed within 48 h after separation. The measurement procedure was detailed in a previous publication (Dulanská et al. 2020). The detection efficiency (*ɛ*) was calculated using Eq. (1):

1 $$\varepsilon = (-0.0001\times {tSIE}^{2 })+(0.1335\times tSIE)+47.82$$

The quenching parameter and the $tSIE$ are crucial for accurate and reliable measurements in scintillation detection. Quenching refers to the reduction of light output caused by interfering substances, which can decrease counting efficiency and lead to underestimation of radioactivity. The main types include chemical quenching, colour quenching, and self-absorption. The quenching parameter quantifies this effect, enabling corrections for accurate radioactivity measurements. The *tSIE* adjusts and normalizes spectra, aligning quenched spectra with reference unquenched spectra for accurate energy response.

### Preparation of samples for ^210^Pb measurements in tobacco and cigarettes

Samples of tobacco and cigarette ash were obtained from the Slovak market. Popular and most commonly smoked tobacco brands were identified through informal communication with 150 smokers in the region, reflecting first-hand accounts and preferences expressed during these discussions. Five brands of tobacco and five brands of cigarettes, identified as popular and most commonly smoked, were selected for comparison. All the identified tobacco and cigarette brands indicated that they were manufactured in the European Union (EU). However, only two tobacco brands, B and E, specified their countries of production as Hungary and Germany, respectively. The companies producing these tobacco and cigarette brands also provided information that, although they were manufactured in the EU, the tobacco was grown in various unspecified countries worldwide. For the overall analysis using both sorbents, tobacco from multiple packs of cigarettes or tobacco per brand was combined and mixed, and an aliquot of 30–50 g was used for testing. To ensure reproducibility, the analyses were repeated three times. Thirty grams of tobacco samples were dried at 105 °C for 24 h. After drying, the tobacco was re-weighed. Subsequently, the tobacco sample was digested in 200 mL of 14.35 mol/L HNO_3_ with constant stirring using a magnetic stirrer at a temperature of 140 °C for nine hours, and 0.2 mL of Pb^2+^ carrier with a concentration of 10 mg/mL was added. After cooling, the sample was filtered and the filter paper was rinsed with 14.35 mol/L HNO_3_. The sample was then evaporated to a volume of approximately 80 mL. The concentration of the samples was subsequently adjusted with deionized water to the final concentration of 8 mol/L HNO_3_.

### Separation of ^210^Pb

A mass of 0.5 g of sorbent (Sr Resin or AnaLig Sr01) was used to prepare the chromatographic columns. The columns were conditioned with 20 mL of 8 mol/L HNO_3_ at a flow rate of 1 mL/min. Then the leachate from the ash, prepared using acid attacks, was applied to the column. After loading, the sorbent was washed with 15 mL of 8 mol/L HNO_3_, followed by an additional wash with 15 mL of deionized water. Lead was then eluted from the column using 15 mL of 9 mol/L HCL, and the fraction was collected in a beaker. The solution was evaporated to near dryness at a temperature of 180 °C, and the tare weight of an empty vial was determined.

The residue was dissolved in 2 mL of 0.05 mol/L HNO_3_, and the solution was quantitatively transferred into the vial. The beaker was rinsed three more times with 1 mL of 0.05 mol/L HNO_3_, and the rinsed volumes were transferred into the same vial. The vial was then weighed again. Aliquots of 100 μL from the solution were taken for Atomic Absorption Spectrometry (AAS) to determine the chemical yield of lead. The chemical yield was measured using an atomic absorption spectrometer (AAnalyst 400/AAS 1100). The vial containing the sample was stored for 50 days (considering the half-life of ^210^Bi is 5.15 days) in a refrigerator. Subsequently, 15 mL of scintillation solution ULTIMA GOLD AB was added, and ^210^Pb was measured using a liquid scintillation spectrometer (TRI CARB 3100TR).

The protocols for the two methods were identical, except for the type of sorbent used. For Method 2, the Sr Resin sorbent from Eichrom was used, while for Method 1, the AnaLig Sr01 sorbent from IBC was utilized based on molecular recognition principles.

### Determination of activities and chemical yields of ^210^Pb

The chemical yield of lead was determined in solutions using Flame-AAS (Perkin-Elmer Model 1100, USA) under standard conditions for lead analysis. Calibration solutions were prepared from CRM Merck CertiPUR Pb 1000 mg/L (Germany). The analysis employed a wavelength of 217 nm with a slit width of 0.7 nm with the deuterium background correction. The calibration range for the analysis was set at 0.2–20 mg/L. Subsequently, 15 mL of scintillation solution ULTIMA GOLD AB was added and then ^210^Pb was measured on a liquid scintillation spectrometer TRI CARB 3100TR. An optimized energy window ranging from 50 to 700 keV was selected to minimize background counts and achieve the Minimum Detectable Activity (MDA). The minimum detectable activity value for ^210^Pb determined using the AnaLig sorbent was 1.11 mBq/g, and for the Sr Resin sorbent it was 1.22 mBq/g. Measurements were conducted using α/β discrimination, allowing for the differentiation between alpha emitters (^210^Po) and beta emitters (^210^Pb and ^210^Bi). Lead fractions from Sr Resin and AnaLig Sr01, mixed with scintillation cocktail, were counted using a TRICARB 3100 TR (PerkinElmer) instrument. Within the investigated quenching interval, ranging from 276 to 564 in *tSIE* units, the detection efficiency (ɛ) varied between 77 and 84% (Fig. 1). In this particular instance, correction for the increase of ^210^Bi is unnecessary (Dulanská et al. 2020). Activity concentration in Bq/g of tobacco was calculated using Eq. (2), where *ɛ* is the detection efficiency, *R* is the chemical yield, ${cpm}_{s}$ is the count per minute of samples, and *m* is the mass of tobacco and cigarettes in dry mass (g):

2 $$A= \frac{{cpm}_{s}}{\varepsilon \times R\times m\times 60} [\text{Bq}/\text{g}]$$

The accuracy of these methodologies was validated using tobacco samples labelled as active, with the addition of certified ^210^Pb (Dulanská et al. 2024). The uncertainties of the measured activities and chemical recoveries were calculated in accordance with the 'Guide to the Expression of Uncertainty in Measurement' (ISO 1995), as referenced by Currie (1999).

### Calculation of annual effective dose for smokers

The results of ^210^Pb activity concentrations in cigarettes and tobacco were recalculated to activity per cigarette, assuming the mass of tobacco in one cigarette to be 0.7 g. The annual effective dose was calculated using Eq. 3:

3 $${H}_{E}=0.22 { \times A}_{c}\times N\times T\times F$$

where ${H}_{E}$ represents the annual effective dose (μSv/y), ${A}_{c}$ is the radionuclide activity per cigarette (Bq/cig.), and $F$ is the effective dose coefficient for ^210^Pb for adult members of the public, which is 1.1 µSv/Bq for inhalation (ICRP 119 2013), $N$ represents the assumed number of cigarettes smoked per day (fixed at 20 cigarettes per day), and $T$ represents the smoking period per year (365 days per year) (Boujelbane et al. 2020). The factor 0.22 indicates that 22% of ^210^Po (and presumably its precursor ^210^Pb) is retained in the smoker's lungs (Kubalek et al. 2016).

## Results and discussion

**Fig. 2:**
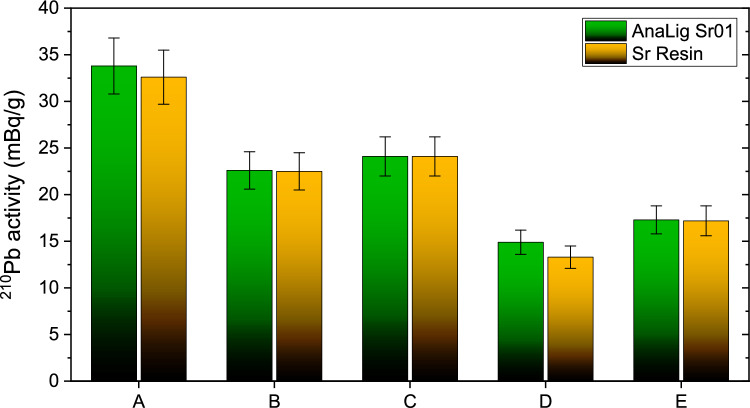
Comparison of ^210^Pb activity concentrations in tobacco samples in Slovakia. For details see text

The results of the ^210^Pb activity concentrations in tobacco and cigarettes sold in Slovakia are presented in Table 1 and Fig. 2.

**Table 1:** Activity concentrations of ^210^Pb in tobacco from commonly smoked brands in Slovakia provided in terms of mBq per gram of dry mass

Type of tobacco	AnaLig Sr01 (Method 1)		Sr Resin (Method 2)	
	R ± U (%)	A ± U (mBq/g)	R ± U (%)	A ± U (mBq/g)
A	99.9 ± 9	33.8 ± 3.0	100 ± 9	32.6 ± 2.9
B	100 ± 9	22.6 ± 2.0	93.4 ± 8.4	22.5 ± 2.0
C	100 ± 9	24.1 ± 2.1	100 ± 9	24.1 ± 2.1
D	100 ± 9	14.9 ± 1.3	100 ± 9	13.3 ± 1.2
E	100 ± 9	17.3 ± 1.5	63.1 ± 5.7	17.2 ± 1.6
Average	100 ± 9	22.5 ± 2.0	91.3 ± 8.2	21.9 ± 2.0

R represents the chemical yield, A is the activity of ^210^Pb per gram of tobacco, and U = k × u(x) represents the total uncertainty expressed at a 95% confidence level, where k = 2 and x is the recovery or the activity

The results of the activity concentrations of ^210^Pb in Slovak tobacco and cigarettes, as determined using both AnaLig Sr01 and Sr Resin methods, show significant variations. The concentrations ranged from 13.3 to 33.8 mBq/g for tobacco and from 16.8 to 28.5 mBq/g for cigarettes. Both methods yielded consistent results, confirming that the protocols were equally effective in separating ^210^Pb. The highest average activity concentration was observed in tobacco A (mean 33.2 ± 3.0 mBq/g), while the lowest was found in tobacco B (mean 14.1 ± 1.3 mBq/g). Despite the slight difference in chemical yields, with AnaLig Sr01 demonstrating a higher average yield of 100% compared to Sr Resin's 91.3%, the activity concentrations remained consistent, indicating that both methods are reliable.

**Fig. 3:**
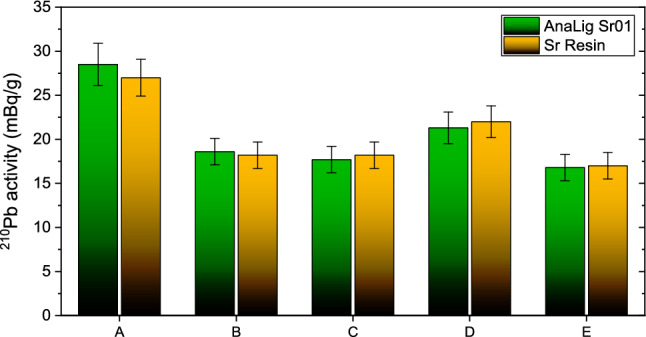
Comparison of ^210^Pb activity concentrations in Slovak cigarette brands. For details see text

**Fig. 4:**
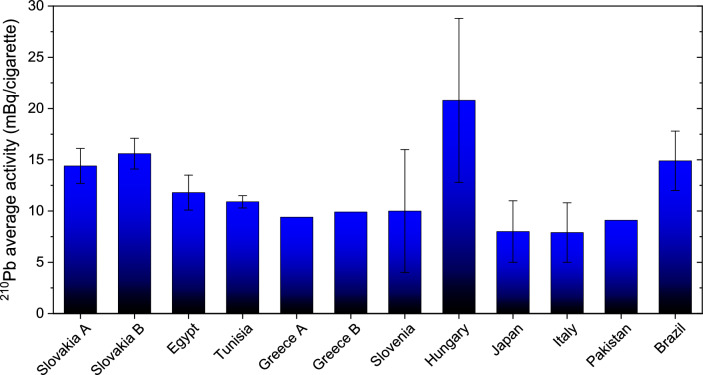
Comparison of the average activity of 210Pb average activity in one 0.7 g cigarette in various countries (see Table 3). Slovakia (**A, B**), Egypt—Din 2021; Tunisia—Boujelbane et al. 2020; Greece—Savvidou et al. 2006; Papastefanou et al. 2009; Slovenia—Kubalek et al. 2016; Hungary—Kovács et al. 2007; Japan—Sakoda et al. 2012; Italy—Desideri et al. 2007, Pakistan—Tahir and Alaamer 2008 2007; Brazil—Peres and Hiromoto 2002

The concentrations of ^210^Pb in cigarettes varied from 16.8 to 28.5 mBq/g, with an arithmetic mean of 20.6 ± 1.7 mBq/g. Cigarettes A showed the lowest average ^210^Pb content at 16.9 ± 1.5 mBq/g while cigarettes A exhibited the highest average content at 27.8 ± 2.3 mBq/g. Results are presented in Table 2 and Figs. 3 and 4.

**Table 2:** Activity of ^210^Pb in cigarettes from commonly smoked brands in Slovakia, provided in terms of mBq per gram of dry mass

Type of cigarettes	Method 1		Method 2	
	R ± U (%)	A ± U (mBq/g)	R ± U (%)	A ± U (mBq/g)
A	98.6 ± 8.8	28.5 ± 2.4	69.6 ± 5.7	27 ± 2.1
B	89.2 ± 8.0	18.6 ± 1.5	87.3 ± 7.8	18.2 ± 1.5
C	83.2 ± 7.4	17.7 ± 1.5	88.4 ± 7.9	18.2 ± 1.5
D	88.2 ± 7.9	21.3 ± 1.8	89.2 ± 8.0	22 ± 1.8
E	87.4 ± 7.8	16.8 ± 1.5	83.6 ± 7.5	17 ± 1.5
Average	89.3 ± 8.0	20.6 ± 1.7	83.6 ± 7.4	20.5 ± 1.7

R represents the chemical yield, A is the activity of ^210^Pb per gram of tobacco, and U represents the total uncertainty

The results of ^210^Pb activity in cigarettes and tobacco were recalculated to reflect activity per cigarette, considering the mass of tobacco in one cigarette to be 0.7 g. The recalculated results were then compared with those of other authors (second column of Table 3). The average activity values for ^210^Pb in cigarettes obtained in the present study were 14.4 ± 1.5 mBq/cigarette and the average values for tobacco were 15.5 ± 1.7 mBq/cigarette When comparing these results with those reported in the literature, the activity values of ^210^Pb in 0.7 g tobacco samples obtained in the present study appear slightly higher than those reported by other authors (Fig. 3). These results closely align with the Brazilian average of 14.9 ± 2.9 mBq/cigarette (Peres and Hiromoto 2002), and are consistent with the worldwide average of 14.88 mBq/cigarette (Boujelbane et al. 2020). Din noted that the activity concentrations of ^210^Pb varied among cigarette brands due to differences in raw tobacco material and manufacturing methods (Din 2021). Interestingly, no significant difference in activity concentrations was observed between imported and local cigarette tobacco brands.

**Table 3:** Experimentally determined radionuclide activity ${A}_{c}$ of ^210^Pb per cigarette (mBq/cig) in cigarettes (mass 0.7 g) from various countries (columns 1 and 2)

References	${A}_{c}$ (^210^Pb) average (mBq/cig.)	${H}_{E}$(µSv/y)	*F* (μSv/Bq)	Equation
Slovakia tobacco This study	15.5 ± 1.7	27.9	1.1	${H}_{E}=0.22 N {A}_{c} F T$
Slovakia cigarettes This study	14.4 ± 1.5	25.5	1.1	${H}_{E}=0.22 N {A}_{c} F T$
Egypt (Din 2021)	11.8 ± 1.7	21.2 ± 2.9	1.2	${H}_{E}=0.22 N {A}_{c} F T$
Tunisia (local) (Boujelbane et al. 2020)	10.9 ± 0.4	19.4 ± 0.8	1.1	${H}_{E}=0.22 N {A}_{c} F T$
Greece (Savidou et al. 2006)	9.4	163	5.6	${H}_{E}=0.75\times 0.5 F {A}_{c} N$
Greece (Papastefanou et al. 2009)	9.9	104.7	1.1	${H}_{E}=0.75 F A {M}_{T}$
Slovenia (Kubalek et al. 2016)	10 ± 6	9*	1.1	${H}_{E}=F A{ M}_{T}$
Hungary (Kovács et al. 2007)	20.8 ± 8	58.7 ± 22.7	1.1	${H}_{E}={F}_{1} {F}_{2} F {A}_{c} N T$
Japan (Sakoda et al. 2012)	8 ± 3	22 ± 9	1.1	${H}_{E}={F}_{1} {F}_{2} F {A}_{c} N T$
Italy (Desideri et al. 2007)	7.9 ± 2.9	163	5.6	${H}_{E}=F{ A}_{c} N$
Pakistan (Tahir and Alaamer 2008)	9.1	64 ± 20	5.6	${H}_{E}={0.5 M}_{T} F A$
Brazil (Peres and Hiromoto 2002)	14.9 ± 2.9		5.6	

Effective annual dose ${H}_{E}$ in µSv/y (column 3), effective dose coefficient *F* (column 4), and equation used in the calculation of annual effective dose (column 5). *A* the activity inhaled by smokers annually (Bq/y); $N$ assumed number of cigarettes smoked per day; $T$ smoking period per year (365 days per year); ${M}_{T}$ annual amount (kg/y) of tobacco consumed; ${F}_{1}$ average transfer factor from tobacco to smoke; ${F}_{2}$ ratio of inhaled smoke to total smoke. Symbol “*” denotes the annual effective dose due to ^210^Pb retained in the lungs after smoking, as determined by Kubalek et al. (2016)

### Determination of annual effective dose for smokers

The committed effective dose coefficients for the inhalation of ^210^Pb and ^210^Po are outlined in Annex B of Publication 68 (ICRP 68 1994) of the International Commission on Radiological Protection (ICRP), derived using the human respiratory tract model from ICRP Publication 66 (ICRP 66 1994). According to ICRP Publication 119 (ICRP 119 2013) recommendations, the effective dose coefficient for ^210^Pb for adult members of the public is 1.1 µSv/Bq for inhalation. To calculate this coefficient, a material of Type M (deposited materials with intermediate rates of absorption into the blood from the respiratory tract) is assumed. In Din (2021), it is noted, however, that this assumption does not align with typical smoking inhalation conditions (31.5 m^3^/h, 0.4 μm active median aerodynamic diameter). The ICRP standard values for Type M materials include an absorption rate and particle size distribution relevant for comparison. For the most accurate estimation of the effective dose for adult smokers, the dose conversion factor calculated using the DCAL software was utilized, providing a value of 1.2 µSv/Bq for ^210^Pb (Schayer et al. 2009). This conversion factor was based on the smoking inhalation conditions mentioned above and an absorption rate of Type M.

When comparing the results of annual effective doses due to ^210^Pb in cigarette tobacco, it is observed that various authors use distinct calculations, conditions, and conversion factors for ^210^Pb. Boujelbane calculated the effective doses per year using the same formula as that used in the present study (Eq. 3) (Boujelbane et al. 2020). In particular, they used an effective dose conversion factor *F* as derived from ICRP 119 (ICRP 119 2013) of 1.1 μSv/Bq for ^210^Pb. Savidou and co-workers postulated that 75% of the ^210^Po (a decay product of ^210^Pb) in cigarettes is present in the smoke (Savidou et al. 2006).

Additionally, it was suggested that ^210^Pb is also present in cigarette smoke, with 50% of the total smoke being inhaled by the smoker. Savidou et al. (2006) utilized a dose coefficient of 5.6 μSv/Bq for ^210^Pb in their calculations. Papastefanou computed the annual effective dose using an equation including a factor of 0.75, indicating that 75% of the radioisotope in cigarette tobacco is found in cigarette smoke, which is then partially inhaled and subsequently deposited in lung tissues (Papastefanou 2009). Kovács and co-workers (Kovács et al. 2007) assumed—as Savidou and co-workers (Savidou et al. 2006)—that 50% of the total smoke is inhaled. However, Kovács and co-workers (Kovács et al. 2007) and Sakoda and co-workers (Sakoda et al. 2012) introduced two additional factors: ${F}_{1}$ representing the average transfer factor from tobacco to smoke and ${F}_{2}$, which is the ratio of inhaled smoke to total smoke. Kubalek and co-workers found in their experiments that after smoking one cigarette, approximately 22% of ^210^Po (and presumably its predecessor ^210^Pb) is retained in the smoker's lungs (Kubalek et al. 2016). This value of 0.22 is therefore included in some studies when calculating annual effective doses (Boujelbane et al. 2020; Din 2021), and it was also used in the present study. Peres and Hiromoto (Peres and Hiromoto 2002) assumed that an individual smokes 20 cigarettes per day and that 10% of the Pb and 20% of the Po are inhaled by primary smokers (UNSCEAR 1982). These authors and Desideri et al. (2007) applied a dose coefficient for adults of 5.6 μSv/Bq for ^210^Pb. Tahir and Alaamer assumed that a smoker inhales approximately 50% of the smoke from a cigarette containing a concentration of ^210^Pb, which was determined experimentally (Tahir and Alaamer 2008). The values of effective doses per year calculated by various authors are summarized in Table 3.

Since different authors have used different formulae to calculate annual effective doses, the methodology used in the present study (Eq. 3) was recalculated using the equations used by other authors, for a direct comparison.

It is very challenging to compare the annual effective doses of ^210^Pb in tobacco, as nearly every author uses a different calculation method, leading to significant variations in results. Depending on the equation used by different authors for calculating the annual effective dose, the values can vary significantly. As shown in Table 3, reported annual effective doses range from as low as 9 µSv/y in Slovenia (Kubalek et al. 2016) to as high as 163 µSv/y in Italy and Greece (Desideri et al. 2007; Savvidou et al. 2006). In the present study, the calculated annual effective doses for Slovakian tobacco and cigarettes were 27.9 µSv/y and 25.5 µSv/y, respectively, demonstrating values within the range reported by other studies. Based on these results, the lowest annual effective doses were obtained using K. Din's method, while the highest were nearly 4.5 times greater using Boujelbane’s method. This underscores the importance of applying consistent formulas and coefficients when presenting annual effective doses.

In general it was found that the daily inhalation of ^210^Po and ^210^Pb due to smoking in Poland, as well as in other nations, is approximately 20 times higher than the daily inhalation of atmospheric ^210^Po and ^210^Pb (Boryło et al. 2022). This indicates that cigarette smoking is the primary source of inhalation of these radionuclides in adult populations worldwide. For those who smoke two or more packs per day of cigarettes with varying radionuclide concentrations, the effective dose due to inhalation of ^210^Po is significantly higher than that due to the intake of ^210^Po from food and water. In general, it was found that the daily inhalation of ^210^Po and ^210^Pb due to smoking in Poland, as well as in other nations, is approximately 20 times higher than the daily inhalation of these radionuclides from atmospheric sources (Borylo et al. 2022). For Polish adults who smoke one pack of cigarettes per day, the annual effective dose from ^210^Po and ^210^Pb due to smoking is approximately 158 µSv, which is a significant contributor to the total annual dose of 309 µSv from all sources, including food, water, and atmospheric inhalation (Borylo et al. 2022). The average annual effective dose due to natural sources of radiation is estimated to be approximately 1.9 mSv, with an uncertainty interval ranging from 1.1 to 3.3 mSv (UNSCEAR 2019). Based on the results of the present study, the contribution of ^210^Po and ^210^Pb from smoking was calculated as approximately 1.33–1.46% of the average dose of 1.9 mSv (UNSCEAR). This indicates that smoking contributes to the exposure to these radioactive elements, slightly increasing the exposure compared to non-smokers. In conclusion, regardless of the type of cigarette or tobacco, smoking adds to the annual effective dose.

## Conclusion

This study presents the results of ^210^Pb activity concentration measurements in tobacco and cigarettes popular in Slovakia. Both extraction methods used demonstrated very similar results, with concentrations per dry mass ranging from 13.3 to 33.8 mBq/g of tobacco. Notably, brand variations were observed, ranging from 14.1 ± 1.3 to33.2 ± 3 mBq/g ^210^Pb concentrations. Such variations were also observed among cigarette brands, where concentrations ranged from 16.8 to 28.5 mBq/g. Based on a typical cigarette mass of 0.7 g, this corresponds to an average ^210^Pb activity of 14.4 ± 1.7 mBq per cigarette. These results align with global averages, although they are slightly higher than some reported values. Annual effective doses for smokers were calculated using different formulae used in the literature, with values for tobacco ranging from 27.9 to 126.7 µSv and for cigarettes from 25.5 to 115.7 µSv. Discrepancies arise from different dose calculation methods employed by various authors. Nevertheless, it is important to not overlook the additional incorporation of ^210^Pb and ^210^Po due to smoking, as the daily inhalation of these radionuclides from smoking has been reported to be significantly higher than from other sources.

## Supplementary Information

Below is the link to the electronic supplementary material.

Supplementary file1 (DOCX 21 KB)

## Data Availability

No datasets were generated or analysed during the current study.
