# Supplementary material for: Determination of activities of 210Pb in Slovak tobacco and cigarettes: a study on radiological risks
Source: Radiat Environ Biophys. 2024 Dec 12;64(1):191–200. doi: 10.1007/s00411-024-01098-9 (PMC11971057; doi:10.1007/s00411-024-01098-9)
Supplement: Supplementary file 1 — Supplementary file1 (DOCX 21 KB) [file 411_2024_1098_MOESM1_ESM.docx]

Highlights

- Investigation of ^210^Pb activity concentrations in tobacco and cigarettes in Slovakia using AnaLig Sr-01 and Sr Resin methods.
- Significant variations in ^210^Pb activities observed in tobacco, ranging from 13.3 mBq/g to 33.8 mBq/g.
- Concentrations in cigarettes ranged from 16.8 mBq/g to 28.5 mBq/g, with an average of 14.4 mBq per cigarette.
- Annual effective doses for smokers ranged from 27.9 µSv to 126.7 µSv for tobacco and from 25.5 µSv to 115.7 µSv for cigarettes.
- Study emphasizes the need for continuous monitoring and regulation due to radiological risks associated with smoking.
